# Country-level welfare-state measures and change in wellbeing following work exit in early old age: evidence from 16 European countries

**DOI:** 10.1093/ije/dyy205

**Published:** 2018-10-01

**Authors:** Sol Richardson, Ewan Carr, Gopalakrishnan Netuveli, Amanda Sacker

**Affiliations:** 1 Research Department of Epidemiology and Public Health, International Centre for Life Course Studies in Society and Health, University College London, London, UK; 2 Institute of Psychiatry, Psychology & Neuroscience, King’s College London, London, UK; 3 Institute for Health and Human Development, University of East London, London, UK

**Keywords:** retirement, work exit, wellbeing, multilevel, Europe, welfare state, social-protection, benefits, Markov Chain Monte Carlo, variance explained

## Abstract

**Background:**

Although the effects of individual-level factors on wellbeing change following work exit have been identified, the role of welfare-state variables at the country level has yet to be investigated.

**Methods:**

Data on 8037 respondents aged 50 years and over in 16 European countries were drawn from the Survey of Health, Ageing and Retirement in Europe (SHARE) and the English Longitudinal Study of Ageing (ELSA). We employed multilevel models to assess determinants of change in wellbeing following work exit, using CASP-12 change scores. After adjusting for institutionally defined route and timing of work exit, in addition to other individual-level variables, we tested country-level variables including welfare-state regime and measures of disaggregated welfare spending to determine their associations with wellbeing change and the proportion of between-country variance explained.

**Results:**

Individuals whose exit from paid work was involuntary or diverged from the typical retirement age experienced declines in wellbeing. Country effects accounted for 7% of overall variance in wellbeing change. Individuals residing in countries with a Mediterranean welfare regime experienced more negative changes in wellbeing, with a difference of –2.15 (–3.23, –1.06) CASP-12 points compared with those in Bismarckian welfare states. Welfare regime explained 62% of between-country variance. National per-capita expenditure on non-healthcare in-kind benefits (services) was associated with more positive wellbeing outcomes.

**Conclusions:**

National expenditure on in-kind benefits, particularly non-healthcare services, is associated with more favourable wellbeing change outcomes following work exit in early old age. Welfare-state effects explain the majority of between-country differences in change in wellbeing.

**Key Messages**

- Compared with retirees, individuals who exit work in early old age via involuntary routes such as unemployment or disability experience declines in wellbeing.
- Welfare regime explained 62% of between-country differences in wellbeing change following work exit in this analysis of 16 European countries, although country effects only contributed to 7% of overall variation in wellbeing change.
- Individuals residing in countries with a Mediterranean welfare regime experience the most negative change in wellbeing.
- Total per-capita social-protection expenditure, and particularly expenditure on non-healthcare services, was associated with more favourable changes in wellbeing after exit from paid work.
- These results have important implications for welfare policy and underscore the importance of provision of welfare services as greater numbers of workers approach retirement age and exit the labour market.

## Background

Work exit or retirement in early old age is an important socially constructed, age-graded transition with significant implications for health and wellbeing.^1^^,^^2^ This transition is growing in importance as the large ‘baby boom’ cohort in developed economies reaches retirement age and places additional strain on existing welfare-state structures.^3^

Wellbeing change following work exit can be positive or negative.^1^^,^^4^ This is influenced by a range of factors at the individual level in addition to national social and organizational policies that create norms regarding the social legitimacy of different routes and timings of exit.^5^^,^^6^ Although country-level institutional determinants of wellbeing have been studied in cross-section,^7^ it has not been investigated whether these influence changes in wellbeing in response to work exit in early old age.

The association between work exit and individual-level wellbeing is influenced by route of exit, age at exit and other factors present at the time of exit. Exit from work via dismissal, permanent sickness or unemployment results in reduced subjective wellbeing and increased psychological distress.^8^^,^^9^ However, rather than the self-reported route of exit, it is suggested that features of work-exit events such as whether they occurred voluntarily or occurred at appropriate times according to social and institutional norms are drivers of these associations.^10–13^

Attempts have been made to define typologies for grouping countries into welfare ‘regimes’ according to how social-protection benefits are granted, their generosity and duration^14^^,^^15^ (Table 1). Differences in cross-sectional wellbeing have been found between welfare regimes.^16^

**Table 1.** Glossary of terms and summary of countries included in the analytic sample by welfare-state regime

Term	Definition	
Hedonic wellbeing^a^	This perspective of wellbeing emphasizes maximization of pleasurable experiences and minimization suffering. This includes not only bodily or physical pleasures, but allows any pursuit of goals or valued outcomes to lead to happiness	
Eudaemonic wellbeing^a^	This perspective emphasizes personal development and realizing one’s potential. Eudemonic wellbeing reflects positive functioning, personal expressiveness and aspects of self-actualization such as autonomy, personal growth, self-acceptance, life purpose, mastery and positive relatedness	
Welfare typology	A scheme used to categorize countries by welfare regime. Various competing typologies exist, with each emphasizing different aspects of welfare states such as social spending, decommodification or ideology	
Welfare regime^b^	Categories of welfare states within a typology. In Esping-Andersen’s view,^15^ welfare regimes arise due to differences in degree of decommodification achieved, social stratification and the private–public mix of welfare provision (see examples below)	
Decommodification^b^	The extent to which individuals and families can maintain a normal and socially acceptable standard of living regardless of their market performance. Conversely, commodification relates to the extent to which workers and their families are reliant upon the market sale of their labour	
Welfare regime	Description^c^	Countries
Bismarckian	Influenced by early social-welfare policies enacted by German chancellor Otto von Bismarck. These policies are distinguished by its ‘status-differentiating’ welfare programmes in which cash benefits are often earnings-related, administered through employers and geared towards maintaining existing social hierarchies. The role of the family in providing care services is also emphasized and the redistributive impact of welfare transfers is minimal	Austria Germany Netherlands France Switzerland Belgium
Mediterranean	Described as ‘rudimentary’ because they are characterized by their fragmented system of welfare provision consisting of diverse income-maintenance schemes with different levels of provision. Reliance on the family and voluntary sector for services is also prominent	Spain Italy Greece
Social democratic	Characterized by universalism in service provision, generous social transfers, a commitment to full employment and income protection, and a strongly interventionist state. The state is used to promote social equality through a redistributive social-security system	Sweden Denmark
Post-Communist	Formerly Communist countries of Central and Eastern Europe share experiences of the collapse of the universalist Communist welfare state followed by social and economic disruption. In recent years, they have shifted towards marketization and decentralization following examples of Liberal welfare states. State provision of welfare services is minimal	Czech Republic Poland Slovenia Estonia
Liberal	State provision of welfare is aimed at proving a minimal safety net; social-protection levels are modest with strict entitlement criteria and recipients are usually means-tested and stigmatized. Private savings and welfare schemes are encouraged through tax incentives	• England

a Adapted from Vanhoutte, 2012.^32^

b Adapted from Esping-Andersen, 1990.^15^

c Adapted from Bambra *et al.*, 2009.^47^

**Figure 1.**
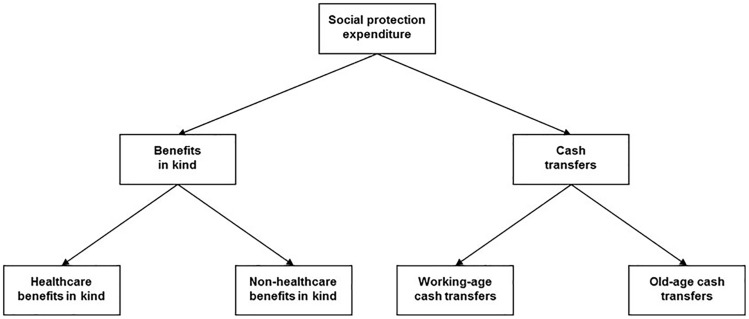
Disaggregation of social-protection expenditure into its primary components.

Earlier welfare typologies, particularly those that employed overall welfare spending measures, manifested a one-sided focus on provision of social insurance^17^^,^^18^ and their failure to differentiate cash transfers from provision of services has been criticized.^19^^,^^20^ A range of comparable quantitative social-spending measures across a number of European countries are available from the OECD Social Expenditure Database (SOCX).^21^ These can be differentiated according to policy area, intended recipients and mode of transfer. As shown in Figure 1, social-protection spending can be categorized into cash transfers and services (in-kind benefits; see Supplementary Table 1, available as Supplementary data at *IJE* online, for definitions)^22^ and then further disaggregated into four primary components: old-age cash transfers (comprising pensions and survivors’ pensions), working-age cash transfers, health benefits in-kind and other service (non-health) expenditure. These components have been shown to be uncorrelated and can be considered distinct dimensions of welfare policy.^23^ Another consideration is how welfare spending is measured. To our knowledge, previous studies on welfare and wellbeing have only considered ‘effort’ measures.^24–26^ These describe the proportion of economic output devoted to social-protection and are expressed as a percentage of gross domestic product (GDP).^27^

Previous work has quantified the degree to which welfare-state factors explain country-level differences in development indicators by partitioning of variance within a multilevel framework.^28^ Multilevel models provide the possibility to estimate both the proportion of variance in an outcome measure explained by country effects vs the proportion attributable to individual characteristics and the proportion of country effects explained by country-level variables.

To our knowledge, no previous study has attempted to quantify country-level influences, in particular welfare-state measures, on wellbeing change in response to work exit in early old age. The present study sought to investigate associations between welfare regime and disaggregated measures of welfare spending with change in wellbeing following work exit, after adjustment for individual-level characteristics. It was hypothesized that respondents in countries such as Scandinavian Social Democratic welfare states with more generous criteria for receipt of benefits^15^ and in countries where spending on social protection is higher experience a more favourable change in wellbeing after exit from paid work. We also aimed to determine whether cash transfers or in-kind benefits had a stronger association with positive wellbeing change, and whether these associations differ when welfare spending is operationalized using measures other than welfare effort.

## Methods

### Analytic sample

The analytic sample was drawn from respondents across 16 countries from Waves 1–5 (2004–13) of the Study of Health, Ageing and Retirement in Europe (SHARE) and Waves 1–6 (2002–13) of the English Longitudinal Study of Ageing (ELSA).^29^^,^^30^ It included participants aged 50 years and over with two or more consecutive waves of observations and who had exited from work since the previous wave. Work exit was defined as a self-reported change in job situation from employed or self-employed at baseline (*t*_0_) to any other state in the following wave (*t*_1_). Where individuals experienced multiple exit events, data on the last event were used. This yielded a total sample of 8548 respondents who had exited from work in the period 2002–13 with wellbeing measures (see Supplementary Figure 1, available as Supplementary data at *IJE* online). Of these, 511 (5.9%) had one or more missing observations for covariates and this yielded a complete sample of 8037 respondents.

### Wellbeing change

Wellbeing was measured using CASP-12 (control, autonomy, self-realization and pleasure)—a shortened version of the validated CASP-19 wellbeing scale (Supplementary Table 2, available as Supplementary data at *IJE* online), previously employed in studies of wellbeing across welfare states.^16^ Its strengths are that it is adapted for individuals in later life and that it provides a global assessment of multiple domains of psychosocial wellbeing by evaluating both hedonic and eudaemonic aspects of wellbeing^31–35^ (see Table 1 for definitions). Exploratory^31^^,^^35^^,^^36^ and confirmatory^35^^,^^37^ factor analyses of CASP-19, in addition to CASP-12,^38^ have shown strong support evidence for a single underlying quality-of-life factor. Wellbeing change was measured using change in CASP-12 scores from *t*_0_ to *t*_1_.

### Route and timing of work exit

Route of exit was defined based on institutional definitions and determined according to type of public benefit received at *t*_1_. This was specified using the benefit categories in SHARE. ELSA responses were mapped onto these (see Supplementary Table 3, available as Supplementary data at *IJE* online). The categories were: (i) disability insurance benefits, (ii) unemployment benefits, (iii) sickness benefits, (iv) social assistance benefits, (v) public early-retirement pension, (vi) public old-age pension and (vii) none of these. When an individual received multiple benefit types, they were assigned to the lowest-numbered category.^39^

We obtained OECD data for ‘typical’ pensionable ages in each country, defined as the earliest point at which an individual can draw full pension entitlements based on a career starting at age 20 with contributions in each year until retirement.^40^ These ages differed for individuals according to their gender and year of exit. Age at exit was determined using self-reported month of exit or, where this was unavailable, the midpoint between *t*_0_ and *t*_1_. Timing of exit was represented using a nominal variable with three categories: (i) work exit >12 months before pensionable age, (ii) work exit within 12 months of pensionable age and (iii) work exit >12 months after pensionable age.

### Covariates

A physical frailty index based on the accumulation of deficits was operationalized using all survey items relating to medically diagnosed conditions, medical symptoms, functional activities and activities of daily living available in both datasets^41–45^ (see Supplementary Note 1, available as Supplementary data at *IJE* online). This scale included 36 items and was calibrated to a range of 0 to 1. Models also adjusted for year of work exit, participation in social activities in the previous month (yes/no), birth outside country of residence (yes/no), partnership status (partnered/non-partnered), country-specific quartile of equivalized non-pension household net wealth and natural logarithm of equivalized gross household income. These variables were selected for inclusion in the fully adjusted model using backward stepwise selection (see Supplementary Note 2, available as Supplementary data at *IJE* online). Individual-level financial variables were purchasing power parity (PPP)-adjusted (see Supplementary Note 3, available as Supplementary data at *IJE* online) and equivalization was performed by dividing by the square root of the household size as per standard OECD methods.^46^

### Welfare regime and country-level social-protection measures

Countries were classified into welfare regimes using the scheme used by Bambra *et al.*^47^ (Table 1). This was based on Ferrera’s welfare-state typology that relates to how social benefits are granted and organized to mitigate labour-market risk and its effects.^47^^,^^48^ Three types of measures of national social-protection spending were obtained from the OECD SOCX database.^21^ In addition to effort, these included ‘emphasis’ (the proportion of government social-protection spending devoted to specific policy areas or benefit type as defined by intended recipients or mode of delivery) and ‘expenditure’ (per-capita government spending by benefit type).^19^ All three measures were then disaggregated into cash and in-kind benefits. Expenditure measures were PPP-adjusted (see Supplementary Note 3, available as Supplementary data at *IJE* online) and further disaggregated into old-age cash benefits, non-old-age cash benefits, in-kind health benefits and non-health in-kind benefits.

### Statistical analysis

Random intercept multilevel models, with individuals nested within countries, were used to account for dependence of observations at the country level. The assumptions of multilevel models include normality of variances, homogeneity of variance and independence of observations at all levels. The small sample of countries available is problematic, however, as standard random intercepts models including fewer than 20–30 level-2 units are likely to yield biased estimates of random-effects parameters.^49^^,^^50^ We therefore employed Bayesian Markov Chain Monte Carlo (MCMC) modelling using Gibbs sampling. This minimizes bias in estimates of variance components even with as few as 10 level-2 units.^51^ MCMC models were run with a monitoring period of 100 000 iterations following a burn-in period of 10 000 iterations to allow model convergence.^52^ Means and standard deviations of sampled model parameters were used to calculate regression coefficients and Bayesian 95% credible intervals (95% CIs). Models adjusted for CASP-12 at *t*_0_ to correct for regression towards the mean.^53^^,^^54^ Data management and descriptive analyses were undertaken in Stata version 14 and MCMC models were run in MLwiN version 3.01.^52^^,^^55–57^

To estimate the extent to which differences in wellbeing change between countries was explained by country-level variables, we fitted a minimally adjusted model for change in CASP-12 (adjusting for CASP-12 at *t*_0_ only). This provided an estimate of the percentage variance explained by country differences. The variance components obtained from this model were used as a baseline for comparison with subsequent models to estimate the percentage of country-level variance explained by groups of country-level variables. The percentage of variation attributable to each level (individual and country) is estimated using the intraclass-correlation coefficient (ICC), defined as ‘the proportion of the variance explained by the grouping structure in the population’.^58^ The ICC of the minimally adjusted model was compared with those obtained from models after adjustment for country-level variables to estimate the proportion of country-level variance explained.

### Analysis strategy

A fully adjusted multilevel MCMC model was fitted with CASP-12 change scores regressed on individual-level variables for the combined sample of SHARE and ELSA respondents. Fully adjusted models were then fitted with the addition of country-level variables. Model 1 added welfare regime, which was fitted as a categorical variable. A further six models (Models 2–7) added groups of variables representing social-protection effort, emphasis and expenditure. Models 2 and 3 fitted total welfare effort, and welfare effort devoted to in-kind and cash benefits, respectively. Model 4 fitted emphasis on in-kind benefits as a percentage of total public social-protection spending. Model 5 fitted total per-capita public expenditure on social-protection benefits, whereas Model 6 disaggregated expenditures into in-kind and cash benefits. In Model 7, expenditures on cash and in-kind benefits were further classified according to whether these were age-related or health benefits. The percentage of variance due to country effects explained by the addition of each set of country-level variables was calculated. Residual plots for level-2 units were generated to show country deviations from the overall mean based on the minimally adjusted model and then the conditional model after adjustment for welfare typology.

## Results

The individual-level characteristics of the analytic sample are shown in Table 2. Supplementary Table 4, available as Supplementary data at *IJE* online, summarizes country-level welfare measures of effort, emphasis and expenditure in 2011 and gives mean values by welfare regime.

**Table 2.** Descriptive statistics of individual-level variables for the analytic sample (*n* = 8037)

		SHARE		ELSA		Combined	
Variable	Categories	n	%	n	%	n	%
Total sample		6031	100	2006	100	8037	100.0
				0			
Route of exit from work	Old-age pension	2952	49.0	601	30.0	3553	44.2
	Disability pension	268	4.4	123	6.1	391	4.9
	Unemployment benefit	314	5.2	25	1.3	339	4.2
	Sickness benefit	106	1.8	6	0.3	112	1.4
	Social Assistance	34	0.6	6	0.3	40	0.5
	Early-retirement pension	590	9.8	0	0.0	590	7.3
	None	1767	29.3	1245	62.0	3012	37.5
							
Age at exit from work	>1 year before pensionable age	2631	43.6	1332	66.4	3963	49.3
	Pensionable age ±1 year	1799	29.8	347	17.3	2146	26.7
	>1 year after pensionable age	1601	26.6	327	16.3	1928	24.0
							
Country-specific quartile of household wealth	1 (poorest)	1090	18.0	228	11.4	1318	16.4
	2	1374	22.8	438	21.8	1812	22.6
	3	1742	28.9	618	30.8	2360	29.4
	4 (wealthiest)	1825	30.3	722	36.0	2547	31.7
							
Participation in activities	Yes	3108	51.5	1104	55.0	4212	52.4
	No	2923	48.5	902	45.0	3825	47.6
							
Partnership status	Married	4957	82.2	1241	61.9	6198	77.1
	Other	1434	23.8	405	20.2	1839	22.9
							
Born abroad	Yes	5537	91.8	1893	94.4	7430	92.4
	No	494	8.2	113	5.6	607	7.6
							
Gender	Male	2900	48.0	938	46.8	3838	47.8
	Female	3131	52.0	1068	53.2	4199	52.3
							
Country	Austria	409	6.8			409	5.1
	Germany	354	5.9			354	4.4
	Sweden	528	8.8			528	6.6
	Netherlands	559	9.3			559	7.0
	Spain	364	6.0			364	4.5
	Italy	340	5.6			340	4.2
	France	533	8.8			533	6.6
	Denmark	512	8.5			512	6.4
	Greece	62	1.0			62	0.8
	Switzerland	418	6.9			418	5.2
	Belgium	653	10.8			653	8.1
	Czech Republic	494	8.2			494	6.2
	Poland	233	3.9			233	2.9
	Slovenia	140	2.3			140	1.7
	Estonia	432	7.2			432	5.4
	England			2006	100.00	2006	25.0
							
Year of exit event	2003	0		215	10.7	215	2.7
	2004	85	1.4	152	7.6	237	3.0
	2005	516	8.6	184	9.2	700	8.7
	2006	352	5.8	153	7.6	505	6.3
	2007	50	0.8	154	7.7	204	2.6
	2008	0		141	7.0	141	1.8
	2009	1428	23.7	288	14.4	1716	21.4
	2010	417	6.9	250	12.5	667	8.3
	2011	754	12.5	340	17.0	1094	13.6
	2012	1975	32.8	129	6.4	2104	26.2
	2013	454	7.5	0		454	5.7
		Median		Median		Median	
Household income	EUR, 2011 PPPs	17 772		18 419		17 954	
							
Frailty index		0.054		0.054		0.054	

Exits from paid work for reasons related to unemployment or disability, and outside typical age windows for retirement, were associated with negative changes in wellbeing. Table 3 shows the results of the multilevel MCMC model for individual-level effects. Both route and timing of work exit were associated with wellbeing change following exit from paid work. The negative CASP-12 change score coefficients indicate that individuals exiting from work via receipt of social assistance (–1.33; 95% CI –2.72, 0.05), unemployment benefit (–1.13; 95% CI –1.66, –0.61), sickness benefit (–2.13; 95% CI –2.97, –1.28) or disability pension (–1.45; 95% CI –1.94, –0.96) experienced more negative wellbeing change compared with those receiving an old-age pension. Exit from work over 1 year before (–0.37; 95% CI –0.63, –0.12) or over 1 year after (–0.46; 95% CI –0.73, –0.19) the relevant year- and gender-specific pensionable age was also associated with more negative CASP-12 change scores.

**Table 3.** Results of a multilevel MCMC model for individual-level determinants of change in wellbeing scores between baseline and follow-up post labour-market exit (*t*_0_ to *t*_1_) in the SHARE and ELSA combined sample (*n* = 8037)

		Combined sample	
Variable	Categories	Coefficient (95% credible interval)	*p*
Route of exit from work	Old-age pension	Ref.	
	Disability pension	–1.45 (–1.93, –0.96)	<0.001
	Unemployment benefit	–1.08 (–1.61, –0.55)	<0.001
	Sickness benefit	–2.07 (–2.92, –1.23)	<0.001
	Social assistance	–1.28 (–2.67, 0.12)	0.036
	Early-retirement pension	0.54 (0.12, 0.97)	0.006
	None	–0.21 (–0.45, 0.03)	0.042
Age at exit from work	>1 year before pensionable age	–0.33 (–0.58, –0.07)	0.006
	Pensionable age ±1 year	Ref.	
	>1 year after pensionable age	–0.44 (–0.71, –0.17)	0.001
Country-specific quartile of household net worth	1 (poorest)	Ref.	
	2	0.85 (0.53, 1.17)	<0.001
	3	1.06 (0.75, 1.37)	<0.001
	4 (wealthiest)	1.38 (1.07, 1.70)	<0.001
Household income	Logged equivalized income	0.26 (0.14, 0.38)	<0.001
Frailty index	Frailty Index	–6.13 (–7.40, –4.86)	<0.001
Participation in social activities	Never	Ref.	
	Yes	0.85 (0.64, 1.05)	<0.001
Partnership status	Partnered	Ref.	
	Non-partnered	–0.26 (–0.49, –0.02)	0.017
Born abroad	No	Ref.	
	Yes	–0.27 (–0.65, 0.10)	0.075
Year of exit event	2003	0.28 (–0.39, 0.95)	0.210
	2004	0.23 (–0.40, 0.85)	0.237
	2005	–0.63 (–1.07, –0.20)	0.002
	2006	–0.43 (–0.90, 0.04)	0.037
	2007	–0.70 (–1.37, –0.04)	0.019
	2008	–0.26 (–1.05, 0.52)	0.255
	2009	–0.30 (–0.64, 0.04)	0.044
	2010	–0.24 (–0.66, 0.19)	0.134
	2011	Ref.	
	2012	–0.16 (–0.49, 0.18)	0.182
	2013	0.40 (–0.09, 0.89)	0.055
Random-effects parameters			
Country		1.13 (0.48, 2.46)	
Individual		19.17 (18.58, 19.77)	

The results of the minimally adjusted model (not shown) found that country of residence accounted for 7% of variance in change in CASP-12 scores following work exit. As shown in Table 4, relative to Bismarckian welfare states, residence in a Mediterranean welfare state was associated with worse wellbeing change following work exit, with an effect size of –2.15 (95% CI –3.23, –1.06) CASP-12 points (Model 1).

**Table 4.** Associations of welfare-state regime and country-level measures of welfare effort, emphasis and expenditure with change in CASP-12 scores following work exit and proportion of between-country differences explained

	Welfare regime	Effort		Emphasis	Expenditure		
Variable^a^	Model 1	Model 2	Model 3	Model 4	Model 5	Model 6	Model 7
Welfare typology							
Bismarckian	Ref.						
Mediterranean	–2.15 (–3.23, –1.06)**						
Social democratic	0.21 (–0.98, 1.43)						
Post-communist	–0.85 (–1.81, 0.15)*						
Liberal	–0.76 (–2.37, 0.78)						
Social-protection spending							
Total public (% GDP)		0.01 (–0.10, 0.10)					
In-kind benefits (% GDP)			0.12 (–0.08, 0.31)				
Cash benefits (% GDP)			–0.07 (–0.19, 0.05)				
In-kind benefits (% public)				0.05 (–0.01, 0.10)			
Total public (EUR 000s)					0.27 (0.02, 0.53)*		
In-kind benefits (EUR 000s)						0.47 (–0.05, 0.97)*	
Cash benefits (EUR 000s)						0.06 (–0.36, 0.52)	
In-kind health benefits (EUR 000s)							–0.15 (–1.43, 1.03)
Other in-kind benefits (EUR 000s)							0.93 (0.00, 2.07)*
Old-age cash benefits (EUR 000s)							0.34 (–0.53, 1.41)
Working-age cash benefits (EUR 000s)							0.13 (–0.76, 1.00)
Country-level variance	0.51	1.18	1.01	0.96	1.00	0.93	1.31
Individual-level variance	19.17	19.18	19.17	19.18	19.15	19.17	19.17
Percent country-level variance	2.57	5.78	4.99	4.77	4.96	4.62	6.38
Percent explained (vs null)	62.11	14.76	26.53	29.66	26.86	31.96	5.99

a Independent effects of country-level welfare-state variables after full adjustment for individual-level variables: route of exit from work, age at exit from work, country-specific quartile of household net worth, logarithm of household income, frailty index, participation in social activities, partnership status, born abroad, year of exit event and CASP-12 at *t*_0_.

** *p* < 0.001; *p < 0.05.

**Figure 2.**
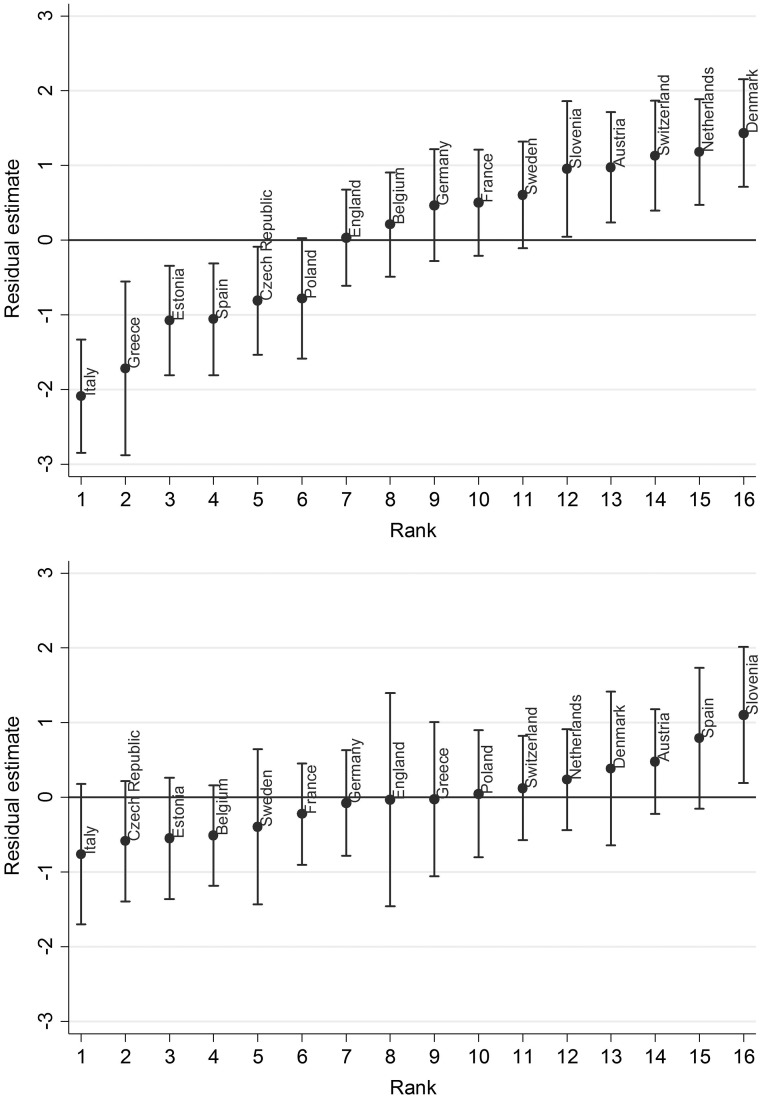
Random intercepts residual plots for level-2 units without adjustment for country-level variables (top, minimally adjusted) and after adjustment for welfare-state regime (bottom, Model 1) showing deviations from the overall mean.

Welfare regime explained 62% of country-level variance in wellbeing change following work exit. Figure 2 shows the effect of adjustment for welfare regime on country-level deviations from the overall mean change in CASP-12. Deviations from the overall mean were attenuated in Model 1 to the extent that, after adjustment for welfare regime, only residence in Slovenia continued to be associated with higher CASP-12 change scores compared with the overall mean.

Models 2–7 (Table 4) show the direct associations of overall and disaggregated measures of effort, emphasis and expenditure with wellbeing change following work exit. Each additional EUR 1000 in total per-capita social-protection expenditure was associated with a 0.27 (95% CI 0.02, 0.53) increase in CASP-12 change scores and accounted for 27% of country-level variance (Model 5). When expenditure was disaggregated into in-kind and cash benefits, we found effect sizes of 0.47 (95% CI –0.05, 0.97) and 0.06 (95% CI –0.36, 0.52) CASP-12 points, respectively, and that these variables accounted for 31.96% of between-country differences (Model 6). Finally, CASP-12 change scores were 0.93 points (95% CI 0.00, 2.07) higher for each EUR 1000 increase in per-capita expenditure on in-kind benefits other than healthcare. No such association was found for expenditure on healthcare services (–0.15; 95% CI –1.43, 1.03) (Model 7).

## Discussion

Work exits occurring over 1 year before or after the typical pensionable age and via unemployment, disability or sickness were independently associated with declines in wellbeing. These adjusted changes in CASP-12 can be greater than or comparable in magnitude to other adverse events such as divorce or separation or diagnosis of a serious physical illness.^59^^,^^60^ Welfare-state regime was strongly associated with wellbeing change following work exit. Expenditure on in-kind benefits, in particular non-healthcare services, was associated with more positive CASP-12 change scores.

Welfare regime explained a higher proportion of between-country differences than any measure of social-protection effort, emphasis or expenditure. Consequently, individuals in Scandinavian Social Democratic welfare states may experience more positive change in wellbeing due to not only higher expenditure on in-kind benefits, but also other institutional factors. These include rules that guide institutional patterns of work exit and individuals’ behaviour and differences in financing mechanisms, extent of benefit coverage and eligibility.^61^ It may be hypothesized that more generous terms of access to cash benefits with longer entitlement periods and universalism of service provision independently buffer against potential negative effects of work exit.

Whereas earlier welfare state typologies focused on the cash-transfer component of welfare spending as the primary differentiator of welfare regimes, welfare services delivered via in-kind benefits have recently received greater attention.^22^ It is argued that maintenance of a socially acceptable standard of living irrespective of individuals’ market performance may also occur through consumption of services independently of market forces (or ‘decommodification’: see Table 1).^20^ Our findings imply that in-kind benefits can play a greater role in buffering against the potential adverse impacts of work exit than cash transfers. Expenditure measures may also be more representative of the actual effects of welfare policies than effort and emphasis measures, as they relate to the direct purchasing power of transfers and value of services rendered.

Expenditure devoted to non-healthcare services had the greatest effect on wellbeing following work exit, and this type of expenditure varies substantially between countries. By contrast, welfare effort devoted to in-kind health-related benefits is relatively similar across developed countries and is unlikely to represent a differentiating feature of welfare-state regimes. Rather, mechanisms of financing and delivery of health services are likely to constitute the primary drivers of national differences in health indicators.^62^ These results underscore the importance of provision of welfare services, such as home help and residential care,^63^ as greater numbers of people in developed economies exit from paid work^3^ (see Supplementary Table 5, available as Supplementary data at *IJE* online, for a summary of cash and in-kind social-protection benefit types by OECD-defined policy area). The results imply that policymakers should prioritize universal provision of non-health services over cash transfers as a more cost-effective means of mitigating potential negative wellbeing consequences of exit. Adverse changes in wellbeing have the potential to negatively impact physiological health and mortality risk.^64^^,^^65^ Health and wellbeing in the post-retirement years of the lifespan will become ever more pertinent as life expectancies increase and retired individuals comprise an ever-increasing proportion of countries’ populations.^66^

This is the first study to address country-level determinants of wellbeing change following work exit and to use a disaggregated spending approach. To date, few studies have considered the associations between welfare spending and wellbeing. Our results agree with those of Eichorn,^24^ which indicate that welfare effort devoted to cash unemployment benefits is not associated with higher life satisfaction among unemployed individuals. Other studies used aggregated country-level wellbeing measures as their outcome and only considered welfare effort. Okulicz-Kozaryn *et al.*^25^ found that overall welfare effort had a positive effect on subjective life satisfaction in cross-section whereas Veenhoven^26^ found no effect.

### Strengths and limitations

The study’s strengths include its large sample size and adjustment for important individual-level determinants of wellbeing change following work exit. Another is its disaggregated spending approach and partitioning of variance components within a multilevel MCMC framework using comparable country-level indicators. This approach presents new avenues for investigating the influence of welfare-state policies across a range of outcome measures.

One assumption of multilevel models is that level-2 units are randomly drawn from a representative sample.^67^ This assumption may have been undermined in our analysis, as the sample of countries available was constrained for pragmatic reasons by their inclusion in SHARE and ELSA and only included OECD member countries with a high level of socio-economic development.^68^ This limits the generalizability of our results to non-European contexts. The analytic sample excluded individuals residing in institutions (e.g. care homes) due to the eligibility criteria for SHARE and ELSA. The fact that the sample comprised individuals who were in employment at baseline and had a mean age of 62.9 years at follow-up implies that the effect on the results was likely to be limited. The sample was likely to have been healthier than the general population of retirees and consequently less likely to require care. Furthermore, the inclusion criteria would not have captured individuals who exited work before the age of 50 years. Sampled respondents would have had similar work histories irrespective of other characteristics such as gender. Finally, negative change in CASP-12 scores attributable to work exit via disability and sickness benefits may be partially due to specific health conditions, which may have been progressive in nature. This potential confounding may not have been fully adjusted for by the frailty measure employed.

## Conclusions

Our findings show that country-level welfare policies explain a large proportion of the variance in wellbeing change between countries and show associations with individual-level wellbeing change following work exit. Expenditure on non-healthcare services had the strongest positive association with wellbeing change.

## Funding

This work was supported by the Economic and Social Research Council (grant numbers ES/J500185/1, ES/J019119/1). The Survey of Health, Ageing and Retirement in Europe data collection has been primarily funded by the European Commission through its fifth and sixth framework programmes (grant numbers QLK6-CT-2001–00360, RII-CT-2006–062193, CIT5-CT-2005–028857). Additional funding was provided by the US National Institute on Aging (grant numbers U01 AG09740-13S2, P01 AG005842, P01 AG08291, P30 AG12815, Y1-AG-4553–01, OGHA 04–064, R21 AG025169) as well as by various national sources. Funding for the English Longitudinal Study of Ageing is provided by the National Institute of Aging (grant numbers 2RO1AG7644-01A1, 2RO1AG017644) and a consortium of UK government departments coordinated by the Office for National Statistics.

## Supplementary Material

Supplementary Data
