# Supplementary material for: Country-level welfare-state measures and change in wellbeing following work exit in early old age: evidence from 16 European countries
Source: Int J Epidemiol. 2018 Oct 1;48(2):389–401. doi: 10.1093/ije/dyy205 (PMC6469302; doi:10.1093/ije/dyy205)
Supplement: Supplementary Data [file dyy205_supplementary_data.docx]

# Online supplement

**Figure S1. Sample flow diagram for the analytic sample of SHARE and ELSA work exit respondents**

**
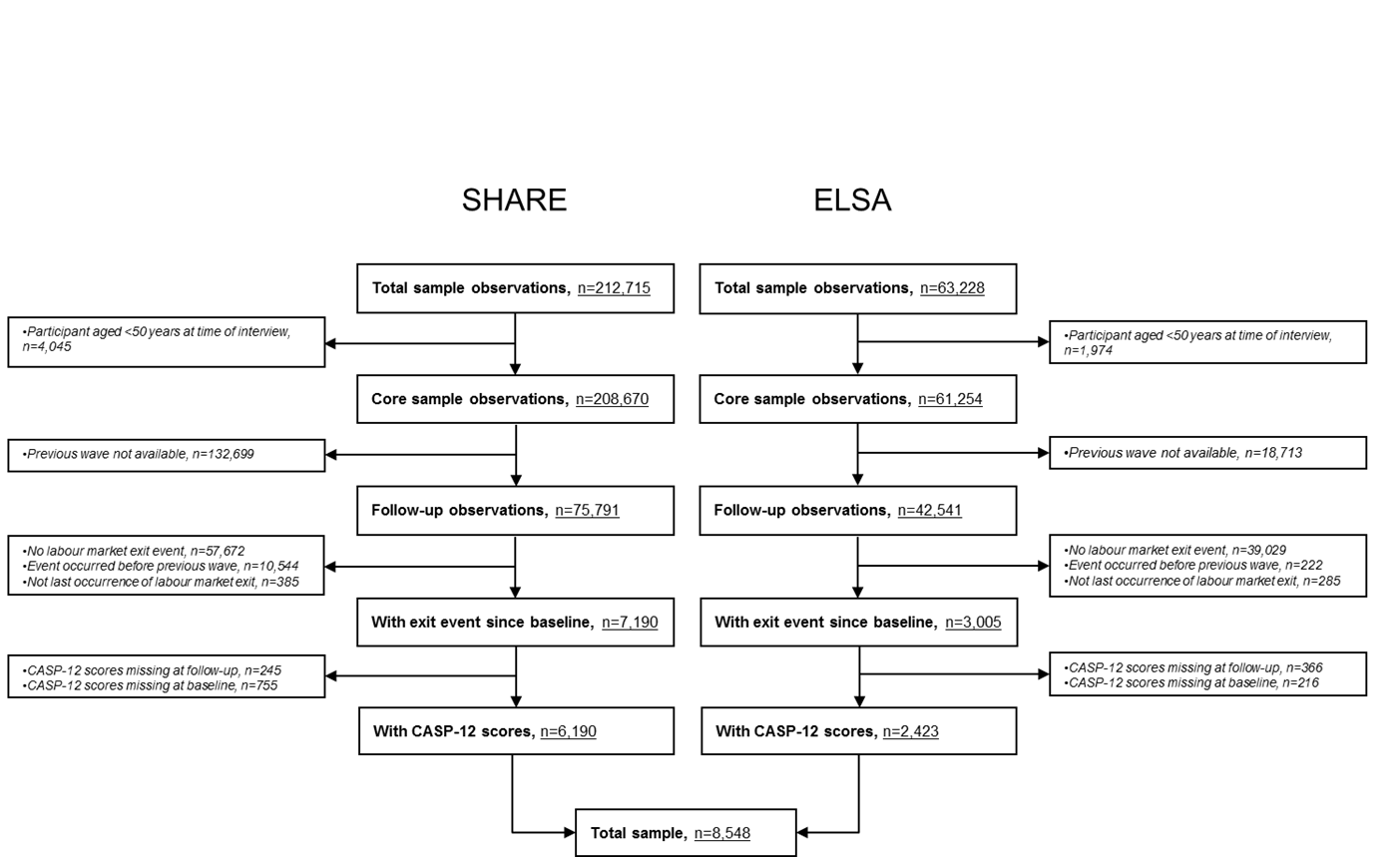
**

| **Table S1. Overview of statistical terms and definitions relating to social protection benefits*** | |
| --- | --- |
| **Term** | **Definition** |
|  |  |
| Social expenditure | The provision by public (and private) institutions of benefits to, and financial contributions targeted at, households and individuals in order to provide support during circumstances which adversely affect their welfare, provided that the provision of the benefits and financial contributions constitutes neither a direct payment for a particular good or service nor an individual contract or transfer. Such benefits are ‘unrequited’: it does not include ‘market transactions’, i.e. payments in return for the simultaneous provision of services of equivalent value cash transfers, and can take the form of cash transfers or direct (“in-kind”) provision of goods and services. |
| Social benefits | Current transfers received by households intended to provide for the needs that arise from certain events or circumstances, for example, sickness, unemployment, retirement, housing, education or family circumstances. |
| Social benefits in kind | These consist of (a) social security benefits, reimbursements, (b) other social security benefits in kind, (c) social assistance benefits in kind; in other words they are equal to social transfers in kind excluding transfers of individual non-market goods and services. |
| Social security benefits in cash | Social insurance benefits payable in cash to households by social security funds; they may take the form of sickness and invalidity benefits, maternity allowances, children’s or family allowances, other dependants’ allowances, unemployment benefits, retirement and survivors’ pensions, death benefits or other allowances or benefits. |

*Adapted from the OECD Glossary of Statistical Terms.

OECD. OECD Glossary of Statistical Terms [Internet]. Paris: OECD Publishing; 2017 [cited 2017 Sep 22].

Available from: <https://stats.oecd.org/glossary/index.htm>

| **Table S2. CASP-19 and CASP-12 scale items and domains** | |  |
| --- | --- | --- |
|  |  |  |
| **Item question** | **CASP Domain** | |
| How often do you think your age prevents you from doing the things you would like to do?^1^ | Control | |
| How often do you feel that what happens to you is out of your control?^1^ |  |  |
| *How often do you feel free to plan for the future? |  |  |
| How often do you feel left out of things?^1^ |  |  |
| How often do you think that you can do the things that you want to do? | Autonomy | |
| How often do you think that family responsibilities prevent you from doing what you want to do?^1^ |  |  |
| *How often do you feel you can please yourself? |  |  |
| *How often do you feel that your health stops you doing the things you want to do?^1^ |  |  |
| How often do you think that shortage of money stops you from doing the things you want to do?^1^ |  |  |
| How often do you look forward to each day? | Self-realisation | |
| How often do you feel that your life has meaning? |  |  |
| *How often do you enjoy the things you do? |  |  |
| *How often do you enjoy the company of others? |  |  |
| How often, on balance, do you look back on your life with a sense of happiness? |  |  |
| How often do you feel full of energy these days? | Pleasure | |
| *How often do you choose to do things you have never done before? |  |  |
| *How often do you feel satisfied with your life? |  |  |
| How often do you feel that life is full of opportunities? |  |  |
| How often do you feel that the future looks good for you? |  |  |
| *Item not included in CASP-12 scale  ^1^Items are reverse-coded to ensure that higher CASP-12 scores were indicative of a higher degree of wellbeing | |  |
|  | |  |

| **Table S3. Comparison of public benefit types in SHARE and ELSA for the specification of a categorical variable representing institutionally-defined route of work exit** | | |
| --- | --- | --- |
|  |  |  |
| **Benefit in ELSA** | **Benefit in SHARE** | **Category*** |
| Incapacity benefit (previously invalidity benefit) | Disability insurance benefits | 1 |
| Severe disablement allowance |  |  |
| Disability living allowance |  |  |
| Industrial injuries disablement benefit |  |  |
| Any other benefit for people with disabilities |  |  |
| Job-seeker's allowance (formerly unemployment benefit) | Unemployment benefits | 2 |
| Statutory sick pay | Sickness benefits | 3 |
| Income support or minimum income guarantee | Social assistance benefits | 4 |
| N/A | Public early retirement pension | 5 |
| State pension | Public old age pension | 6 |

*The categorical variable for route of work exit according to type of public benefits received at t_1_ was generated using benefit

categories in SHARE. ELSA responses were harmonised with these as shown. Respondents receiving multiple benefit types were categorised using the hierarchy proposed by Robroek et al., 2013 (39) and assigned to the lowest-numbered category shown above.

For example, a respondent receiving both disability insurance benefits and a public early retirement pension would be placed in

Category 1.

| **Table S4. Social protection effort, emphasis and expenditure by country and welfare regime (2011)** | | | | | | | | | |
| --- | --- | --- | --- | --- | --- | --- | --- | --- | --- |
| **Welfare regime** | **Country** | **Total public (% GDP)** | **Total public* (EUR per capita)** | **Effort** | | **Emphasis** | | **Expenditure** | |
|  |  |  |  | **In-kind benefits (% GDP)** | **Cash benefits (% GDP)** | **In-kind benefits (% public)** | **Cash benefits (% public)** | **In-kind benefits (EUR per capita)** | **Cash benefits (EUR per capita)** |
| Bismarckian | Austria | 26.1 | 7603 | 8.3 | 17.8 | 31.8 | 68.2 | 2415 | 5188 |
|  | Germany | 23.9 | 6792 | 10.1 | 13.8 | 42.1 | 57.9 | 2858 | 3934 |
|  | Netherlands | 20.9 | 6433 | 9.9 | 11.0 | 47.4 | 52.6 | 3048 | 3385 |
|  | France | 29.6 | 7311 | 11.1 | 18.5 | 37.4 | 62.6 | 2735 | 4576 |
|  | Switzerland | 17.7 | 6414 | 7.6 | 10.1 | 43.0 | 57.0 | 2760 | 3654 |
|  | Belgium | 27.9 | 7596 | 10 | 17.9 | 35.8 | 64.2 | 2719 | 4877 |
|  | *Mean* | *24.4* | *7025* | *9.5* | *14.9* | *39.2* | *60.8* | *2756* | *4269* |
| Mediterranean | Spain | 25.4 | 5477 | 8.8 | 16.6 | 34.7 | 65.3 | 1899 | 3578 |
|  | Italy | 26.9 | 6322 | 7.8 | 19.1 | 29.0 | 71.0 | 1833 | 4488 |
|  | Greece | 25.7 | 4525 | 8.0 | 17.7 | 31.2 | 68.8 | 1414 | 3111 |
|  | *Mean* | *26.0* | *5441* | *8.2* | *17.8* | *31.5* | *68.5* | *1715* | *3726* |
| Social democratic | Sweden | 24.6 | 7126 | 13.3 | 11.3 | 54.2 | 45.8 | 3863 | 3264 |
|  | Denmark | 26.9 | 7722 | 13.5 | 13.4 | 50.1 | 49.9 | 3865 | 3857 |
|  | *Mean* | *25.8* | *7424* | *13.4* | *12.4* | *52.0* | *48.0* | *3864* | *3560* |
| Post-communist | Czech Republic | 19.5 | 3702 | 6.9 | 12.6 | 35.5 | 64.5 | 1314 | 2388 |
|  | Poland | 19.1 | 2804 | 5.4 | 13.7 | 28.3 | 71.7 | 793 | 2012 |
|  | Slovenia | 23.1 | 4371 | 7.3 | 15.8 | 31.7 | 68.3 | 1384 | 2987 |
|  | Estonia | 16.1 | 2551 | 5.3 | 10.8 | 32.7 | 67.3 | 833 | 1717 |
|  | *Mean* | *19.5* | *3357* | *6.2* | *13.2* | *32.2* | *67.8* | *1081* | *2276* |
| Liberal | England | 22.2 | 5413 | 11.2 | 11.0 | 50.3 | 49.7 | 2723 | 2690 |

*All per capita expenditure measures are PPP-adjusted and benchmarked against the relative price level for actual individual consumption in Germany in 2011.

| **Table S5. Types of social protection programme by OECD policy area and expenditure type*** | | | |
| --- | --- | --- | --- |
|  |  | **Social protection benefit type** | |
|  |  | **Benefits in kind** | **Cash transfers** |
|  |  |  |  |
| **Policy area** | Old age | Residential care Home help services Other benefits in kind | Pension Early retirement pension Other old age cash benefits |
|  | Survivors | Funeral expenses Other benefits in kind | Pension Other cash benefits |
|  | Incapacity | Residential care Home help services Other benefits in kind | Disability pensions Paid sick leave Other cash benefits |
|  | Health | Healthcare services | N/A |
|  | Family | Day care Other benefits in kind Home help services | Family allowances Maternity and parental leave Other cash benefits |
|  | Unemployment | Other benefits in kind | Unemployment compensation Severance pay Early retirement pension (for labour market reasons) |
|  | Housing | Housing assistance Other benefits in kind | N/A |
|  | Other | Social assistance Other benefits in kind | Income maintenance Other cash benefits |
| *Adapted from The Social Expenditure database: An Interpretive Guide.  OECD. The Social Expenditure database: An Interpretive Guide. Paris: OECD Publishing; 2007 (63).  Available from: http://stats.oecd.org/oecdstatdownloadfiles/oecdsocx2007interpretativeguide_en.pdf | | | |

# Supplementary notes

1) The physical frailty index was based on the deficit accumulation model, for which a standard method for determining degree of frailty in a clinical context has been proposed by Searle et al. (1). This has been implemented in a number of panel studies including SHARE (2,3,4,5) and ELSA (6).

Rockwood and Mitnitski (7) and Ferrucci et al. (8) have concluded that such frailty scales are strongly predictive of risk of mortality, institutionalisation and adverse health events when 30 or more variables are included, and that estimates become unstable particularly when 10 or fewer are included (1). In addition, items included should be representative of an individual’s health status over as wide a range of functional domains as possible and not saturate at too early an age (i.e. deficits included in the scale must not be near-universal at too young an age) (3). This is particularly important when applying scales to data from the general population and across wider age ranges. One key assumption of the deficit accumulation model, with regards to its relationship with chronological age, is that deficits accumulate over time at a non-linear ‘characteristic’ rate for each individual (7).

Items were selected with the objective of including at least 30 items covering as wide a range of functional domains as possible (7). The index was operationalised using all self-reported items relating to medically-diagnosed conditions, medical symptoms, functional activities and activities of daily living previously included in studies of frailty indices in SHARE (3) and ELSA (6). The final scale included 36 items.

While the scale initially included 37 items, 27.7% of responses in ELSA had missing values for diagnosis of a hip or femoral fracture. This item was therefore removed from the scale as performed by others (7,9) in previous studies. Furthermore, grip strength measures were not considered for inclusion in the scale as these were not available in all waves of ELSA. This could potentially have resulted in a large loss of sample size and statistical power if this measure were included. The table below lists the full range of items included in the scale.

A change in frailty score from 0 to 1 was associated with a difference in CASP-12 change scores from t_0_ to t_1_ of -6.13 (95% CI:-7.40, -4.86, p<0.001) when the fully-adjusted model was fitted for the combined analytic sample (n=8037) (see Table 3). When the same models were run for SHARE (n=6031) and ELSA data (n=2006) separately, the effect sizes were -7.40 (95% CI:-9.00, -5.79, p<0.001) and -4.02 (95% CI:-5.92, -2.12, p<0.001) respectively.

| **Items used to specify a physical frailty scale in SHARE and ELSA** | |
| --- | --- |
|  |  |
| **Variable** | **Categories** |
| ***Medically diagnosed conditions*** |  |
| Myocardial infarction | 1=yes, 0=no |
| Hypertension | 1=yes, 0=no |
| Stroke | 1=yes, 0=no |
| Diabetes or elevated blood sugar | 1=yes, 0=no |
| Chronic Obstructive Pulmonary Disease | 1=yes, 0=no |
| Arthritis | 1=yes, 0=no |
| Osteoporosis | 1=yes, 0=no |
| Cancer | 1=yes, 0=no |
| Parkinson's Disease | 1=yes, 0=no |
| Cataracts | 1=yes, 0=no |
| ***Medical symptoms*** |  |
| Problem sleeping or restlessness | 1=yes, 0=no |
| Difficulty seeing objects at distance | 1=yes, 0=no |
| Difficulty seeing objects at arm's length | 1=yes, 0=no |
| ***Difficulties with functional activities*** |  |
| Walking short distances (100 metres/100 yards) | 1=yes, 0=no |
| Sitting for long periods (≥2 hours) | 1=yes, 0=no |
| Standing up from sitting down | 1=yes, 0=no |
| Climbing several flights of stairs | 1=yes, 0=no |
| Climbing one flight of stairs without resting | 1=yes, 0=no |
| Stooping, kneeling or crouching | 1=yes, 0=no |
| Extending arms above shoulders | 1=yes, 0=no |
| Pulling or pushing large objects | 1=yes, 0=no |
| Carrying or lifting heavy objects (≥5kg/≥10lbs) | 1=yes, 0=no |
| Picking up a small coin from a table | 1=yes, 0=no |
| ***Difficulties with activities of daily living (ADLs)*** |  |
| Dressing (including shoes and socks) | 1=yes, 0=no |
| Walking across a room | 1=yes, 0=no |
| Bathing or showering | 1=yes, 0=no |
| Eating | 1=yes, 0=no |
| Getting in or out of bed | 1=yes, 0=no |
| Using the toilet (including getting up or down) | 1=yes, 0=no |
| Using a map to navigate in a strange place | 1=yes, 0=no |
| Preparing a hot meal | 1=yes, 0=no |
| Shopping for groceries | 1=yes, 0=no |
| Making telephone calls | 1=yes, 0=no |
| Taking medication | 1=yes, 0=no |
| Work in the home or garden | 1=yes, 0=no |
| Managing money | 1=yes, 0=no |

The histogram below shows the distribution of frailty scores in the combined analytic sample.


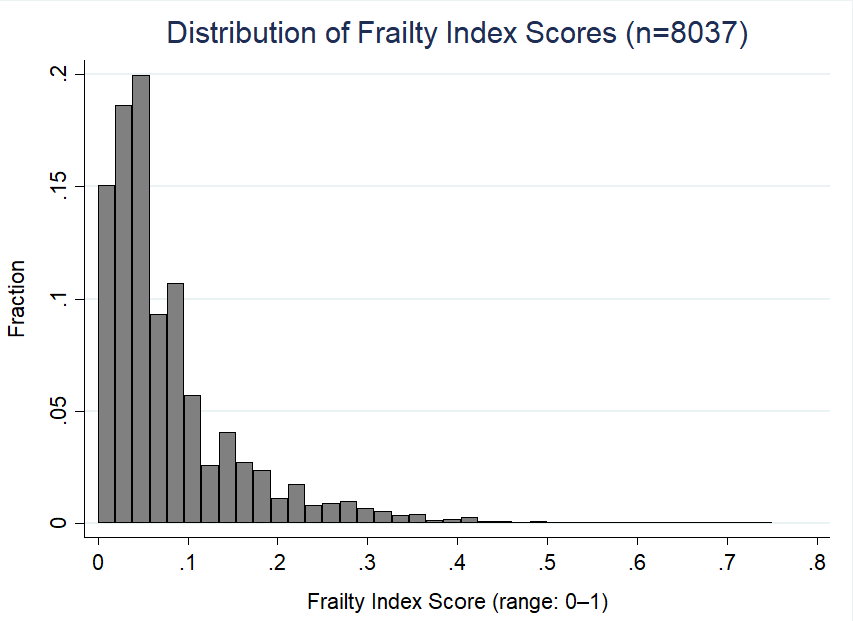


The table below shows the mean (95% CI) and median values of the index for the combined sample, for each dataset (SHARE and ELSA), and for each country in the analytic sample.

| **Mean and median values of the frailty index by dataset and by country** | | |
| --- | --- | --- |
| **Dataset** | **Mean (95% CI)** | **Median** |
|  |  |  |
| Combined | 0.081 (0.079, 0.083) | 0.054 |
| SHARE | 0.080 (0.078, 0.082) | 0.054 |
| ELSA | 0.082 (0.078, 0.086) | 0.054 |
| **Country** |  |  |
|  |  |  |
| Austria | 0.080 (0.072, 0.087) | 0.054 |
| Germany | 0.077 (0.070, 0.084) | 0.061 |
| Sweden | 0.068 (0.062, 0.074) | 0.054 |
| Netherlands | 0.071 (0.065, 0.077) | 0.054 |
| Spain | 0.089 (0.080, 0.099) | 0.054 |
| Italy | 0.073 (0.066, 0.081) | 0.054 |
| France | 0.083 (0.076, 0.090) | 0.054 |
| Denmark | 0.067 (0.061, 0.073) | 0.047 |
| Greece | 0.088 (0.065, 0.110) | 0.054 |
| Switzerland | 0.059 (0.054, 0.064) | 0.047 |
| Belgium | 0.085 (0.079, 0.092) | 0.061 |
| Czech Republic | 0.085 (0.078, 0.092) | 0.061 |
| Poland | 0.117 (0.105, 0.129) | 0.088 |
| Slovenia | 0.090 (0.078, 0.101) | 0.074 |
| Estonia | 0.103 (0.096, 0.111) | 0.081 |
| England | 0.082 (0.078, 0.086) | 0.054 |

References

1. Searle SD, Mitnitski A, Gahbauer EA, Gill TM, Rockwood K. A standard procedure for creating a frailty index. *BMC Geriatr* 2008;8:24.

2. Romero-Ortuno R, Walsh CD, Lawlor BA, Kenny RA. A Frailty Instrument for primary care: findings from the Survey of Health, Ageing and Retirement in Europe (SHARE). *BMC Geriatr* 2010;10:57.

3. Harttgen K, Kowal P, Strulik H, Chatterji S, Vollmer S. Patterns of frailty in older adults: comparing results from higher and lower income countries using the Survey of Health, Ageing and Retirement in Europe (SHARE) and the Study on Global AGEing and Adult Health (SAGE). *PLoS One* 2013;8(10):e75847.

4. Romero-Ortuno R. The Frailty Instrument for primary care of the Survey of Health, Ageing and Retirement in Europe predicts mortality similarly to a frailty index based on comprehensive geriatric assessment. *Geriatr Gerontol Int* 2013;13(2):497–504.

5. Romero-Ortuno R, Soraghan C. A Frailty Instrument for primary care for those aged 75 years or more: findings from the Survey of Health, Ageing and Retirement in Europe, a longitudinal population-based cohort study (SHARE-FI75+). *BMJ Open* 2014;4(12): e006645.

6. Marshall A, Nazroo J, Tampubolon G, Vanhoutte B. Cohort differences in the levels and trajectories of frailty among older people in England. *J Epidemiol Community Health* 2015;69(4):316–21.

7. Rockwood K, Mitnitski A. Frailty in relation to the accumulation of deficits. *J Gerontol A Biol Sci Med Sci* 2007;62(7):722–27.

8. Ferrucci L, Guralnik JM, Studenski S, Fried LP, Cutler GB Jr, Walston JD. Designing randomized, controlled trials aimed at preventing or delaying functional decline and disability in frail, older persons: a consensus report. *J Am Geriatr Soc* 2004;52:625–34.

9. Romero-Ortuno R, Kenny RA. The frailty index in Europeans: association with age and mortality. *Age Ageing* 2012;41(5):684–89.

2) A manual likelihood-ratio-test-based backward stepwise selection procedure, based on Collett’s recommended method (1), was used to determine which independent variables would be included in the final model in addition to CASP-12 at t_0_. The significance level for removal from the model was p=0.1. Below is a list of individual-level variables considered for inclusion in the final model. Asterisked (*) variables were dropped as part of the selection procedure.

- Year of work exit
- Route of exit from work (disability insurance benefits/unemployment benefits/sickness benefits/social assistance benefits/public early retirement pension/public old age pension/other)
- Age at exit from work (>1 year before pensionable age/pensionable age ±1 year/>1 year after pensionable age)
- Frailty index
- Participation in social activities in the previous month (yes/no)
- Birth outside country of residence (yes/no)
- Partnership status (partnered/non-partnered)
- Country-specific quartile of equivalised non-pension household net wealth
- Natural logarithm of equivalised gross household income
- Gender (male/female)*
- Highest last-known level of education (ISCED-97 category) at t_1_ (primary (0 and 1)/secondary (2, 3 and 4)/tertiary (5 and 6)/other or still in education)* (2,3)
- Occupational level (ISCO-88 category) at t_0_ (elementary manual (8 and 9)/skilled manual (6 and 7)/skilled non-manual (3, 4 and 5)/professional (1 and 2))* (4)
- Part-time employment at t_0_ (<30 hours/week) (yes/no)* (5)
- Housing tenure (outright ownership/ownership with outstanding mortgage repayments/renting or other)*
- Effort-reward ratio* (6)

References

1. Collett D. Modelling Survival Data in Medical Research. London: Chapman and Hall; 1991.

2. UNESCO. International Standard Classification of Education: ISCED 1997. Paris: UNESCO; 2006.

3. Schneider S. The Application of the ISCED-97 to the UK’s Educational Qualifications. Mannheim: MZES; 2008.

4. ILO. International Standard Classification of Occupations (ISCO-88). Geneva: International Labour Office; 1990.

5. Bastelaer A, Lemaître G, Marianna P. The definition of part-time work for the purpose of international comparisons. Labour Market and Social Policy Occasional Papers–No. 22. Paris: OECD; 1997.

6. Reinhardt JD, Wahrendorf M, Siegrist J. Socioeconomic position, psychosocial work environment and disability in an ageing workforce: a longitudinal analysis of SHARE data from 11 European countries. *Occup Environ Med* 2013;70(3):156–63.

3) All financial variables including country-level expenditure measures were expressed in PPP-adjusted Euros using the relative price level for actual individual consumption in Germany in 2011 as the baseline. Data were obtained from the Prices and Purchasing Power Parities database. For countries not part of the Euro currency area during the study period, adjusted financial variables were converted to Euros using year-average nominal exchange rates provided by EUROSTAT. Comparisons between years were made by extrapolating annual consumer price index (CPI) inflation terms for all consumer items (i.e. PPP(2009) *= [Relative price level for actual individual consumption relative to Germany in 2011]*[CPI inflation between 2009 & 2010]*[CPI inflation between 2010 & 2011]*[Nominal exchange rate in 2009]*).

**Acknowledgements**

We would like to thank Dr Elisabetta Trevisan of the Marco Fanno Department of Economics and Management at the University of Padua for her guidance on the calculation of purchasing power parity conversion factors for the harmonisation of financial variables.

**Funding statement**

This work was supported by the Economic and Social Research Council (grant numbers ES/J500185/1, ES/J019119/1).

The Survey of Health, Ageing and Retirement in Europe data collection has been primarily funded by the European Commission through its fifth and sixth framework programs (grant numbers QLK6-CT-2001-00360,RII-CT-2006-062193, CIT5-CT-2005-028857). Additional funding was provided by the U.S. National Institute on Aging (grant numbers U01 AG09740-13S2, P01 AG005842, P01 AG08291, P30 AG12815, Y1-AG-4553-01, OGHA 04-064, R21 AG025169) as well as by various national sources.

Funding for the English Longitudinal Study of Ageing is provided by the National Institute of Aging (grant numbers 2RO1AG7644-01A1, 2RO1AG017644) and a consortium of UK government departments coordinated by the Office for National Statistics.

**Conflict of interest:** None declared
